# Low T-cell reactivity to TDP-43 peptides in ALS

**DOI:** 10.3389/fimmu.2023.1193507

**Published:** 2023-07-21

**Authors:** Swetha Ramachandran, Veselin Grozdanov, Bianca Leins, Katharina Kandler, Simon Witzel, Medhanie Mulaw, Albert C. Ludolph, Jochen H. Weishaupt, Karin M. Danzer

**Affiliations:** ^1^ Neurology, University Clinic, University of Ulm, Ulm, Germany; ^2^ Institute of Experimental Cancer Research, Medical Faculty, University of Ulm, Ulm, Germany; ^3^ German Center for Neurodegenerative Diseases (DZNE), Ulm, Germany; ^4^ Neurology, Medical Faculty Mannheim, Heidelberg University, Heidelberg, Germany

**Keywords:** amyotrophic lateral sclerosis, T cells, TDP-43, autoantibody, autoantigen

## Abstract

**Background:**

Dysregulation of the immune system in amyotrophic lateral sclerosis (ALS) includes changes in T-cells composition and infiltration of T cells in the brain and spinal cord. Recent studies have shown that cytotoxic T cells can directly induce motor neuron death in a mouse model of ALS and that T cells from ALS patients are cytotoxic to iPSC-derived motor neurons from ALS patients. Furthermore, a clonal expansion to unknown epitope(s) was recently found in familial ALS and increased peripheral and intrathecal activation of cytotoxic CD8^+^ T cells in sporadic ALS.

**Results:**

Here, we show an increased activation of peripheral T cells from patients with sporadic ALS by IL-2 treatment, suggesting an increase of antigen-experienced T cells in ALS blood. However, a putative antigen for T-cell activation in ALS has not yet been identified. Therefore, we investigated if peptides derived from TDP-43, a key protein in ALS pathogenesis, can act as epitopes for antigen-mediated activation of human T cells by ELISPOT and flow cytometry. We found that TDP-43 peptides induced only a weak MHCI or MHCII-restricted activation of both naïve and antigen-experienced T cells from healthy controls and ALS patients. Interestingly, we found less activation in T cells from ALS patients to TDP-43 and control stimuli. Furthermore, we found no change in the levels of naturally occurring auto-antibodies against full-length TDP-43 in ALS.

**Conclusion:**

Our data suggests a general increase in antigen-experienced T cells in ALS blood, measured by *in-vitro* culture with IL-2 for 14 days. Furthermore, it suggests that TDP-43 is a weak autoantigen.

## 1 Introduction

Innate and adaptive immune responses accompany the degenerative process in the fatal neurodegenerative disease amyotrophic lateral sclerosis (ALS) (1). The complex dynamics that parallel disease onset and progression include not only cells of the central nervous system (CNS), but also peripheral immune cells: infiltration of peripheral immune cells into the brain and spinal cord has been well-documented in ALS patients and different animal models of the disease (2–5). However, whether they contribute to human disease, and how, is not completely understood. T cells have gained particular attention in ALS, as they represent a significant proportion of the peripheral cells that infiltrate the CNS of ALS patients, their phenotype is altered in ALS, and they bear high therapeutic potential. CD4^+^ T cells have been shown to be protective in ALS, but their abundance and regulatory capacities are diminished in ALS patients (6); the number and functionality of regulatory T cells (T_reg_) correlates negatively with ALS progression and suggests a novel avenue for therapeutic interventions (7, 8). In addition, cytotoxic CD8^+^ T cells were recently also implicated in ALS pathogenesis by the demonstration that they can selectively kill motor neurons (9). In that study, the interaction of CD8^+^ T cells with motoneurons relied on the MHC-class I molecules’ association with self-antigens and their interaction with T-cell receptors. However, the underlying autoantigens remain unknown, which might be critical for further understanding of the involvement of T cells and their different subsets in ALS. In line with this, more recent studies found a clonal expansion of cytotoxic CD8^+^ T cells to an unknown antigen in familial ALS with mutation in the *SETX* gene (10) and an increase in activated (HLA-DR^+^) CD8^+^ T cells in the blood and CSF of patients with sporadic ALS (11). Therefore, it has been suggested that a common ALS-associated antigen like TAR DNA-binding protein 43 (TDP-43) or SOD1 might trigger T-cell mediated autoimmune processes (12). Interestingly, in a similar manner it was recently shown that T cells in Parkinson’s Disease (PD) selectively recognize autoantigens generated from alpha-synuclein, a protein which plays a major role in the etiology of the disease (13). Several proteins linked to ALS share the pathological features of alpha-synuclein in PD, including generation of toxic oligomers, high molecular-weight aggregation, aberrant dynamics of proteostasis and subcellular mislocalization. Among these proteins, TDP-43 stands out by being the histopathological hallmark of the disease, as well as by its linkage to genetic forms of ALS (14–18). In motoneurons, TDP-43 pathology results in mislocalization, aggregation and aberrant processing of the protein (19). We therefore hypothesized that TDP-43 might be a so-far unidentified autoantigen contributing to motoneuron demise in ALS. To test this hypothesis, we investigated the reactivity of T cells from healthy controls and ALS patients to ten TDP-43-derived peptides.

## 2 Methods

### 2.1 Human samples

All participants provided informed written consent in accordance with the declaration of Helsinki and approved by the Ethics Committee of Ulm University. The probands were recruited at Universtitäts- and Rehabilitationskliniken (RKU) Ulm. ALS patients were diagnosed by the El-Escorial criteria and considered sporadic if a negative family history was reported. Participants with confounding conditions affecting the immune system were excluded from the study. Participants were not selected/excluded based on clinical parameters like disease progression or disease duration. Venous blood was collected using standard procedure with the Monovette™ blood drawing system (Sarstedt) in K3-EDTA tubes. PBMCs were purified from whole blood by density-gradient centrifugation over Histopaque (Sigma) at 500 g (30 min) and plasma was collected for the quantification of autoantibodies.

### 2.2 Peptide design

TDP-43 peptides were designed using the online database ‘Immune Epitope Database (IEDB) and Analysis Resource’ (www.iedb.org) (20, 21). Initially all linear epitopes of TDP-43 with MHC-I and MHC-II binding specificity detected in all assays with human as host were selected. Subsequently the ‘Epitope Analysis Resource’ was used for T-cell epitope prediction for TDP-43 protein individually for MHC-I and MHC-II binding with the ANN tool as prediction method and cross-referenced for with the SYFPEITHI database (22). Only peptides with high binding affinity (IC50<50nM with the ANN tool) were retained. Five peptides with appropriate length for MHC-I presentation and five with length for MHC-II presentation were selected based on wide HLA binding range, high binding affinity and overlap with protease cleavage sites (predicted with ExPASy PeptideCutter v.3.0 (23), ProteomicsDB (24) and MEROPS v12.1 (25)) and commercially synthesized (ThermoFisher Scientific).

### 2.3 *In vitro* expansion of PBMCs

PBMCs for *in vitro* expansion were cultured in RPMI 1640 (Sigma) supplemented with 5% human AB serum (Sigma), and 1% penicillin-streptomycin (P/S) (Sigma). For stimulation, PBMCs were seeded in a concentration of 2 x 10^6^ cells/well in a 24-well tissue culture plate (Sarstedt). To study the response of naïve T cells to TDP-43 peptides, PBMCs were primed with pools of MHC-I or MHC-II peptides, each consisting of five peptides at a 1 µg/mL concentration each. Phytohemagglutinin (PHA) (Sigma) was used as a positive control (5 µg/mL); MOG (CTL, CTL-PM-MOG) and actin (CTL, CTL-PM-ACTS) were used as negative controls (5 µg/mL). In order to study the response of memory T cells, PBMCs were cultured without any peptide stimulus for 14 days and then stimulated with individual TDP-43 peptides during ELISPOT assay or for flow cytometry. IL-2 (BioLegend) was added at 20 ng/mL every three days over a period of 14 days in order to promote T-cell proliferation.

### 2.4 ELISPOT

All steps for ELISPOT assay were carried out under sterile conditions in duplicates and following suppliers’ instructions. Immobilon^®^-P PVDF membranes (Merck) were activated with methanol, washed with sterile water and coated with an IL-5 antibody (TRFK5, Mabtech) and an IFN-γ antibody (1-D1K, Mabtech) at 5 µg/mL. Plates were then blocked with RPMI1640 media with 5% human AB serum and 1% (P/S). PBMCs were seeded at 10^5^ cells/well with 10 µg/mL of individual peptides and the plates were incubated for 36 h at 37 °C. Cells and supernatant were then discarded, plates washed six times with PBS-T and IL-5 and IFN-γ detected with biotinylated antibodies (5A10, Mabtech and 7-B6-1, Mabtech), streptavidin-HRP (Mabtech, 3310-9) and Vectastain ABC-AP (APC) staining solution (Vector Laboratories) as per suppliers’ instructions. Spots were detected on an Immunospot Reader (CTL). A response to an antigen was defined as a spot-forming activity over the DMSO (vehicle) control for the specific proband and assay (IL-5 or IFN- γ).

### 2.5 Monocyte culture and ELISA

Monocytes were isolated from PBMCs with CD14-magnetic beads (MACS) as described previously (26), cultured in RPMI1640, 10% FCS, 1% (P/S) and stimulated with TDP-43 peptides for 24 h. IL-6 release in cell culture media was detected with ELISA as described before (26) with the ELISA Max Deluxe Set Human IL-6 kit (BioLegend).

### 2.6 Flow cytometry

*In-vitro* expanded PBMCs for flow cytometry analysis were harvested at 14 days *in vitro* (DIV), seeded in tissue culture plates at a density of 10^6^ cells/well and re-stimulated with 10 µg/mL of individual TDP-43 peptides per sample. After 24 h of incubation, the cells were blocked with human TruStain Fc block (BioLegend) and stained with antibodies specific for CD3 (1:50, BioLegend, 300431, Pacific Blue), CD4 (1:100, BioLegend, 300508, PE), CD8 (1:100, BioLegend, 344722, APC), CCR7 (1:20, BioLegend, 353226, PE-Cy7), CD45RA (1:20, BioLegend, 304106, FITC) and HLA-DR (1:50, BioLegend, 307630, PerCP-Cy5.5). An unstained control was used to measure auto-fluorescence of the cell suspension and the following isotype controls; Mouse IgG1, κ-PB (1:50, BioLegend, 400151), Mouse IgG1, κ-PE (1:100, BioLegend, 400112), Mouse IgG1, κ-APC (1:100, Invitrogen, 17-4714-82), Mouse IgG2a, κ- PE/Cy7 (BD Biosciences, 1:20, 400232),Mouse IgG2a, κ-FITC(1:20, BioLegend, 400208), Mouse IgG1, κ-PerCP/Cy5.5 (1:50, BioLegend, 400150), were used for the measurement of non-specific binding of the antibodies. The samples were analyzed using BD LSR II (BD Biosciences). Singlets were selected based on the forward and side scatter and defined as the total PBMCs. CD3^+^ cells were selected from the total singlets. CD4^+^, CD8^+^, double-negative, double-positive, central memory, effector memory and TEMRA subsets were selected from the total CD3^+^ cells gate. HLA-DR expression was quantified in each T-cell subtype.

### 2.7 Quantification of naturally occurring anti-TDP-43 autoantibodies in plasma and serum

Naturally occurring autoantibodies in plasma were quantified by a luminescence-based IgG-capture assay. To this end, diluted plasma samples (1:50 in PBS) were mixed 1:1 with the filtered (0.22 µm) supernatant from HEK293 cells overexpressing a fusion protein of full-length human wild-type TDP-43 and humanized *Gaussia* luciferase described before (27). The resulting complex of TDP-43-Luciferase and anti-TDP-43 autoantibodies was then captured on a protein-G coated 96-well plate by incubation at RT for 4 h with shaking (450 rpm) with subsequent washing (5x 200 µL PBS) and quantified by measurement of the luciferase activity on a Victor X3 system as described before (27, 28). This method has several advantages over the use of indirect ELISA with recombinant TDP-43: it can simultaneously capture autoantibodies to TDP-43 monomers, oligomers and high-molecular weight oligomers; the TDP-43 substrate is produced by mammalian cells and is captured ‘in solution’, thus reflecting more closely physiological conditions. Unspecific binding to *Gaussia* luciferase was controlled by including supernatant from HEK293 cells expressing *Gaussia* luciferase alone as a negative control. Recombinant anti-TDP-43 antibody was used when establishing the assay as a positive control. Alpha-synuclein antibodies were quantified in serum with the same assay with a previously described fusion protein of human wild-type alpha-synuclein and the humanized *Gaussia* luciferase (28).

### 2.8 Data analysis

Statistical analysis was performed with Prism 9.3.1 (GraphPad Software, La Jolla, CA) and R 3.6.3. (29). Gaussian distribution was tested with D’Agostino and Pearson omnibus normality test, Shapiro–Wilk normality test, and Kolmogorov–Smirnov normality test. Statistical significance was tested with Mann-Whitney U-test, Welch’s t test, Wilcoxon matched-pairs signed ranks test, one-way and two-way ANOVA with Sidak’s or Bonferroni correction. All statistical tests were two-tailed. ELISPOT data was analyzed using the average spot forming cells per sample. Flow Cytometry data was analyzed using FACS Diva 8.0.1. ELISA data was analyzed using 5-parameter log-logistic curve fitting (BMG Labtech).

## 3 Results

**Figure 1:**
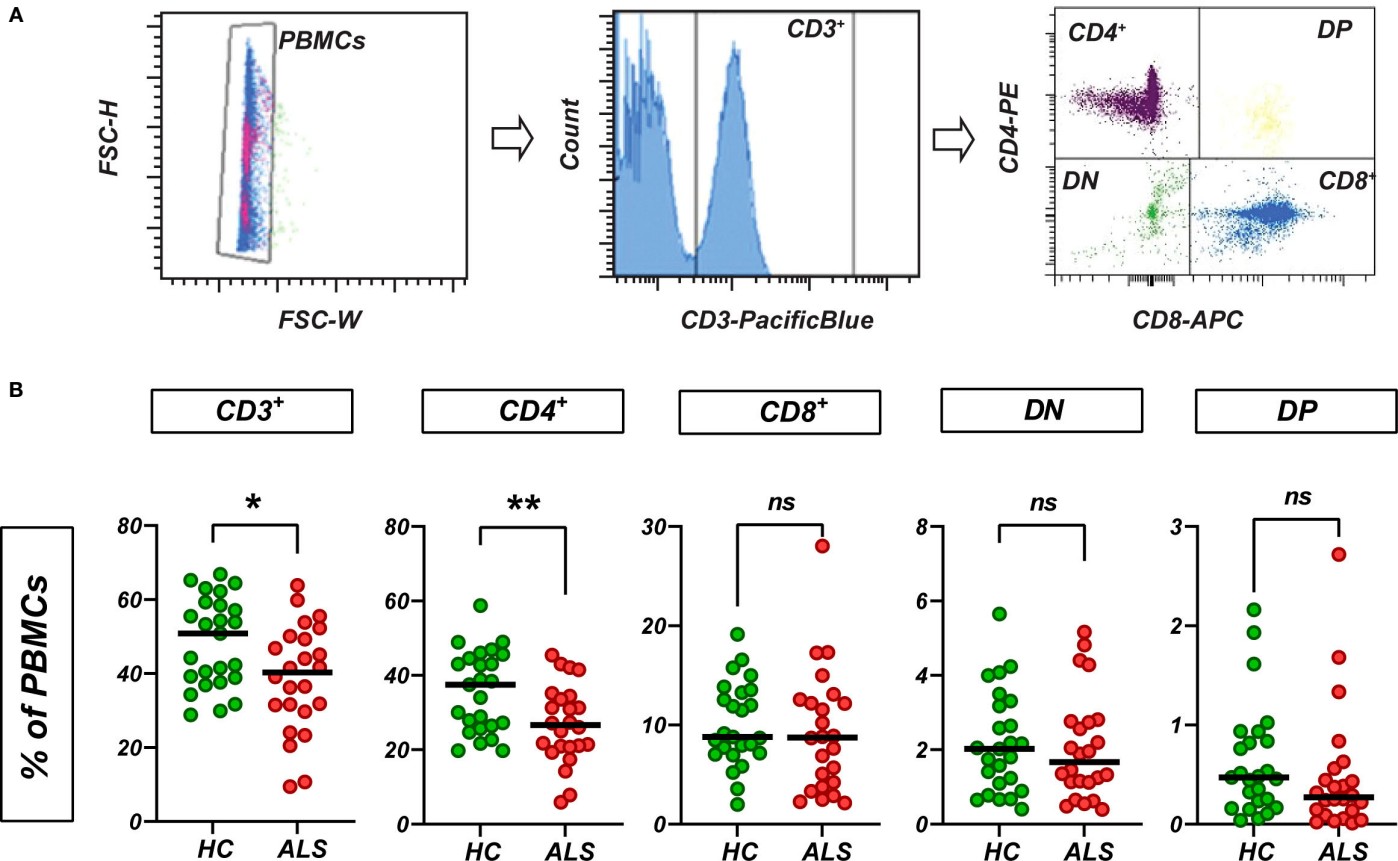
Circulating T cells in ALS and healthy control blood. **(A)** T cells were quantified based on the CD3 expression. CD3^+^ subsets were quantified as % of PBMCs. **(B)** Relative abundance (as % of PBMCs) of CD3^+^, CD4^+^, CD8^+^, double-negative (DN) and double-positive (DP) T cells in PBMCs from healthy controls and ALS patients (n=25/24). Lines: median. *p<0.05, **p<0.01, Mann-Whitney U-test. ns, not significant.

To obtain a comprehensive picture of the circulating T cells in ALS patients, we performed flow cytometry with peripheral blood mononuclear cells (PBMCs) from 24 ALS patients and 25 age-matched healthy controls (**Supplementary Table 1**). To determine the net frequencies of T cells in PBMCs, we selected CD3^+^ cells and quantified cell numbers as percent from the total singlet PBMCs (**Figure 1A**). In line with previous observations (30–32), we found a net decrease of the relative numbers of CD3^+^ cells, which was driven by a net decrease of CD4^+^ cells, while net CD8^+^, double-negative (DN) and double-positive (DP) T cells were unchanged in ALS (**Figure 1B**). We did not observe statistically significant differences in the relative numbers of CD4^+^, CD8^+^, DN and DP cells when quantified as percent of all CD3^+^ cells (**Supplementary Figure 1**).

**Figure 2:**
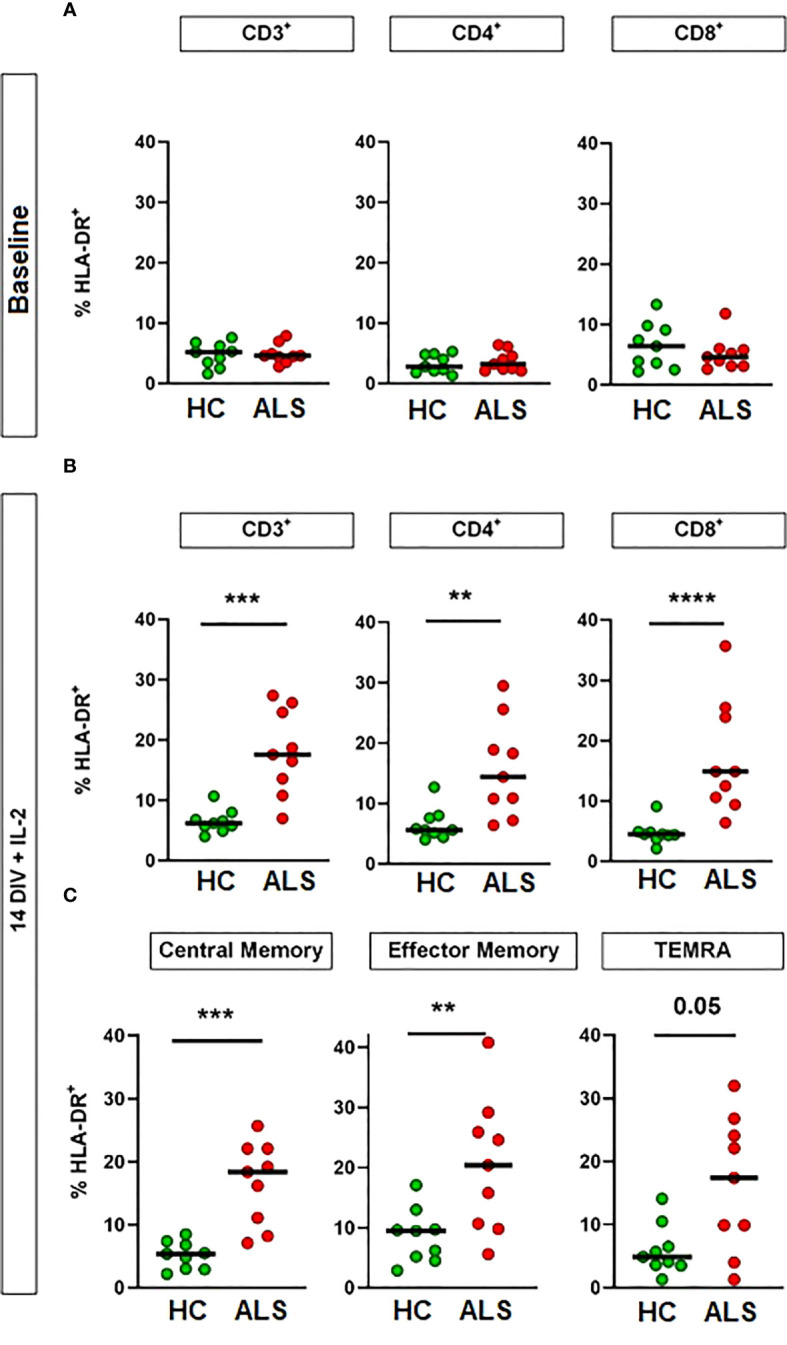
Increased activation of ALS T cells by culturing with IL-2. Activation of T cells was assessed by flow cytometry for HLA-DR expression. Percentage of HLA-DR^+^ cells in circulating CD3^+^ T cells and T-cell subsets at sample collection **(A)** and after 14 days *in vitro* (DIV) with IL-2 supplementation **(B, C)**. N = 9 (healthy controls)/9 (ALS patients). Lines: median. **p<0.01, ***p<0.001, ****p<0.0001, Mann-Whitney U-Test. DIV: Days *in vitro*. IL-2: 20 ng/mL.

Both CD4^+^ and CD8^+^ T cells can be activated upon recognition of their cognate antigen. To investigate whether there is an increased abundance of antigen-activated T cells in ALS, we analyzed T-cell activation by flow cytometry for HLA-DR expression, a well-established marker of T-cell activation, before and after expansion with IL-2. We chose HLA-DR, as it specifically represents the late stage of T-cell activation, i.e. it allows to quantify how many of the circulating T cells have already come to an initial contact with their cognate antigen. In addition, both CD4^+^ and CD8^+^ T cells express HLA-DR after activation. While T-cell activation was comparable on baseline between healthy controls and ALS patients, we found a significant increase of HLA-DR-expressing T cells in ALS, affecting both CD4^+^ and CD8^+^ cells, upon culturing and expansion of T cell for 14 days *in vitro* with IL-2 (20 ng/mL) (**Figures 2A, B**, n=9/9; **Supplementary Table 2**). Furthermore, T-cell activation was observed in all three main T cell memory types: central memory, effector memory and TEMRA, while effector memory T cells showed the strongest *in-vivo* expansion, as expected (**Figure 2C**, **Supplementary Figures 2**, **3**). This change suggests an increased presence of antigen-experienced T cells that express IL2R after antigen-mediated activation in ALS.

**Figure 3:**
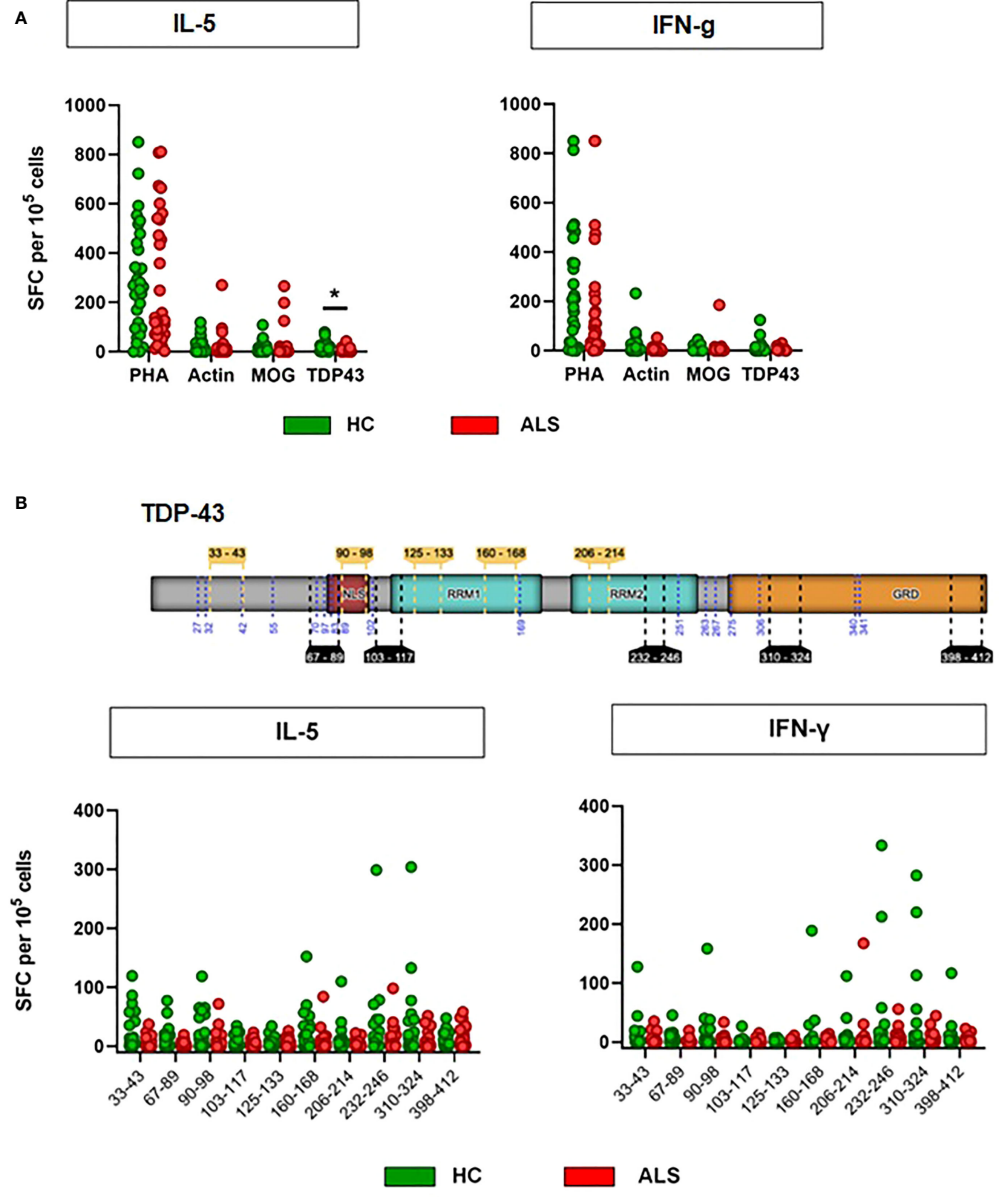
Antigen-restricted activation of T cells by TDP-43 peptides. **(A)** Activation of T cells was assessed with ELISPOT for IL-5 and IFN-γ after stimulation with PHA, MOG peptides, Actin peptides or TDP-43 peptides. N = 32 (healthy controls)/33 (ALS patients). Lines: median. *p<0.05, Mann-Whitney U-Test. PHA: Phytohaemagglutinin, MOG: Myelin oligodendrocyte glycoprotein. **(B)** Antigen-restricted activation of T cells by *single* TDP-43 peptides. Schematic representation of TDP-43 and the peptides used for the stimulation of T cells. Activation of T cells was assessed with ELISPOT for IL-5 and IFN-γ. N = 32 (healthy controls)/33 (ALS patients) (24/27 for MOG). *p<0.05, Mann-Whitney U-Test, Wilcoxon matched-pairs signed ranks test. Start and end positions in the canonical TDP-43 protein sequence (NP_031401.1) indicated for the five peptides intended for MHC-I presentation (yellow inlets above the protein sequence) and for the five peptides indeded for MHC-II presentation (black inlets below the protein sequence). TDP-43 domains indicated: ‘NLS’, nuclear localicaton signal; ‘RRM1’ & ‘RRM2’, RNA-recognition domain 1 and 2; ‘GRD’, glycine-rich domain.

**Figure 4:**
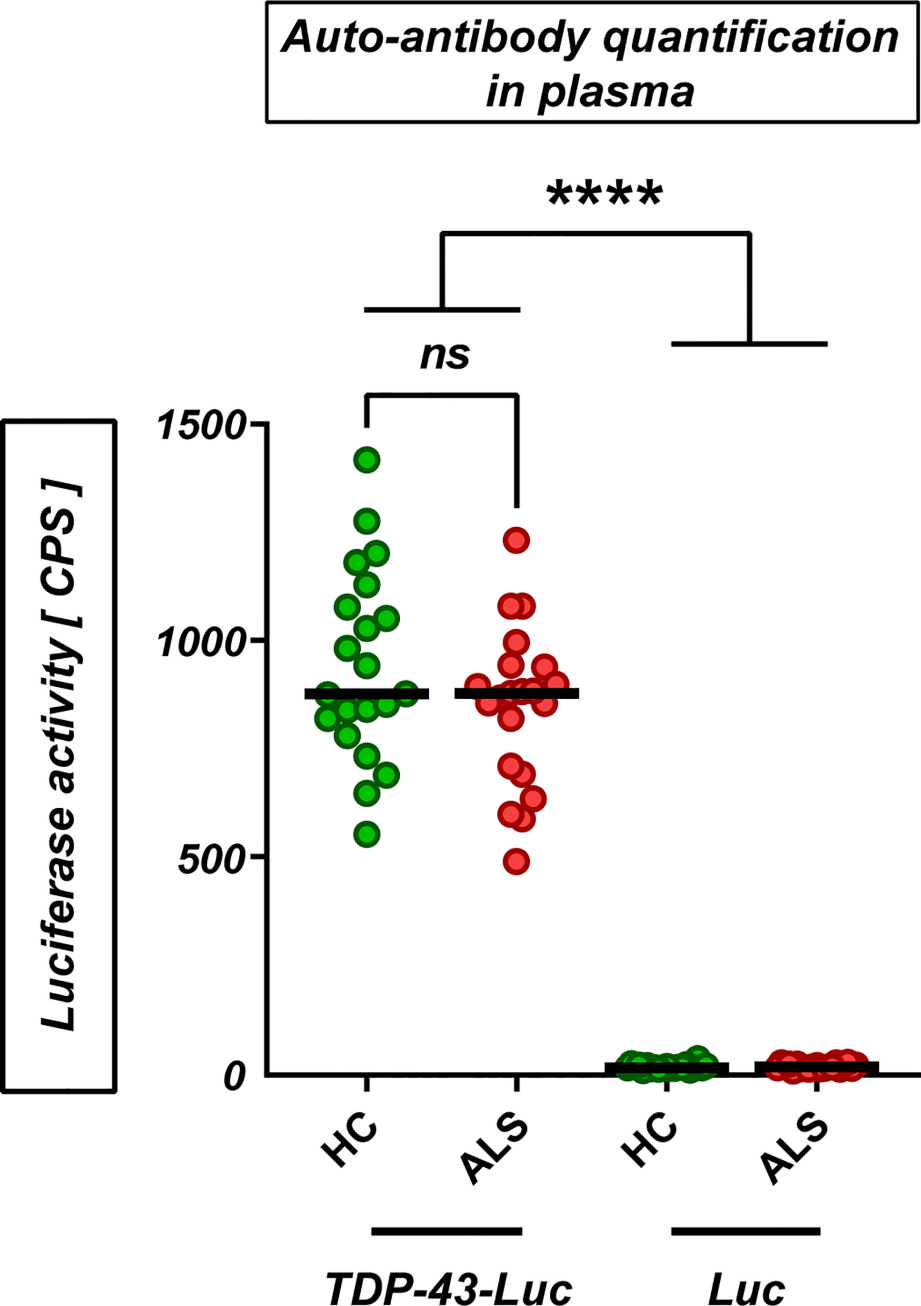
Naturally-occurring auto-antibodies against TDP-43 in plasma. Levels of autoantibodies against TDP-43 in plasma from healthy controls and ALS patients (HC/ALS: n=21/21; 61.6 ± 8.7/62.1 ± 7.9 y; f/m: HC: 9/12, ALS: 9/12) were measured by capturing of TDP-43-luciferase complexes with plasma auto-antibodies (*‘TDP-43-Luc’*) on a protein-G coated plate. Luciferase alone as negative control (*‘Luc’*). *ns’*: not significant (Mann-Whitney U-test); ****p<0.0001 (One-Way ANOVA with Sidak’s correction).

We then hypothesized that the TDP-43 protein, which is prone to misfolding and aggregation and at the same time represents the major neuropathological marker in almost all ALS patients, acts as a T-cell activating antigen in ALS. To test this hypothesis, we designed a set of 10 peptides derived from the wild-type human TDP-43 (UniProt:Q13148) (**Figure 3B**, **Supplementary Table 3**). Most peptide sequences were chosen to be flanked by at least one predicted or known protease cleavage site, thus imitating the natural processing of TDP-43. Five of the peptides had a length of 9-11 amino acids, which is required to fit in the peptide cleft of the MHC class I complex, while the other five peptides have a length of 15 amino acids, fulfilling the minimal length for MHC class II-mediated antigen presentation. MHC-I and MHC-II peptides were distributed evenly across the sequence of TDP-43 and were designed for high binding affinity (IC50<50nM) to a wide range of HLA alleles. All main domains of TDP-43 were covered by the peptides, including the ALS mutational hotspot in the C-terminal glycine-rich domain (**Figure 3B**). First, we excluded that TDP-43 peptides result in unspecific activation of PBMCs, as they did not activate isolated peripheral blood monocytes at three different concentrations (**Supplementary Figure 4**). We then used the peptides to stimulate PBMCs from ALS patients and healthy controls in culture (n=33/32, mean disease duration 49 ± 38 months, mean ALSFRS-R 32 ± 11, **Supplementary Table 4**) and quantified the T-cell response by ELISPOT for IL-5 and IFN-γ (**Figure 3A**, **Supplementary Table 5**). Stimulation of PBMCs with the potent mitogen PHA (positive control) induced a strong, antigen-unspecific activation of PBMCs, with a slight, non-significant decrease in activation of PBMCs from ALS patients (**Figure 3**, **Supplementary Table 5**). As expected, PBMCs from ALS patients and healthy controls responded weakly to peptides from the autoantigens actin or myelin oligodendrocyte glycoprotein (MOG, data available in 24/27 cases), with no significant difference between healthy controls and ALS patients. The response to TDP-43 peptides was comparable or lower to the response to MOG and actin in magnitude; however, we found significantly lower responses by IL-5-producing T cells in ALS. The percentage of responders (persons with a response higher than the spontaneous SFC-activity in the DMSO vehicle control) was comparable to that for actin and less in ALS for both IL-5 (>25%) and IFN-γ producing cells (>30%) (Fisher’s exact: 0.43 (IL-5), 0.46 (IFN- γ)). The median response in the responder groups was significantly lower in ALS in IL-5 producing, but not in IFN-γ producing cells (**Supplementary Figure 5**). Taken together, this data suggests a possibly lower CD4^+^-mediated T-cell response to TDP-43 in ALS. We then analyzed in detail which TDP-43 peptides elicit the response of PBMCs (**Figure 3B**, **Supplementary Tables 6**, **7**). We observed that all TDP-43 peptides contributed to the activation of PBMCs, with a slight tendency for a stronger reaction to the peptides closer to the C-terminus. In addition, the weak antigenic response was observed both to peptides designed for MHC-I and for MHC-II-restricted activation. In general, we observed more often responses by IL-5 producing T cells than by IFN-γ producing cells, but the responses by IFN-γ producing cells were stronger in amplitude. The IL-5 response correlated positively with the IFN-γ response per individual and peptide, which can be attributed to individual differences in T-cell activity or to differences in the binding affinity of the peptides to the different HLA alleles expressed by the different persons. The response to most peptides was decreased in ALS, both in IL-5 producing and in IFN-γ producing cells. Moreover, we did not find any difference between the abundance of naïve and of antigen-experienced T cells reactive to TDP-43 (**Supplementary Figure 6**). As a complementary measure of autoimmunity, we also quantified the abundance of naturally-occurring autoantibodies against full-length wild-type TDP-43 monomers and oligomers (**Figure 4**) in blood plasma. We found low, but measurable levels of TPD-43-specific autoantibodies, but no difference between healthy controls and ALS patients. Levels of TDP-43 autoantibodies were significantly higher than the negative control in this assay (****p<0.0001, luciferase alone) and higher than the levels of naturally occurring antibodies to alpha-synuclein (**Supplementary Figure 7**); however, they were much lower than the levels of antibodies to alpha-synuclein in one person with known high blood concentrations of alpha-synuclein antibodies (**Supplementary Figure 7**), as measured with a similar assay.

## 4 Discussion

In the present study, we found elevated levels of activated T cells in ALS blood after *in vitro* expansion with IL-2. By assaying HLA-DR expression as activation marker, we captured the frequency of circulating T cells, which have already come in contact with their cognate antigen. HLA-DR is a late T-cell activation marker, which is up-regulated after other common activation markers (CD25, CD69), which could not be assessed in this study. Similar to some previous reports (30–32), we found a significant decrease of total CD3^+^ and net CD4^+^ cells in ALS. This decrease in CD3^+^, driven by a decrease in CD4^+^ cells, might reflect an increased infiltration of T cells into the ALS CNS, as reported previously (33). Apart from this, we found comparable composition of T cells in healthy controls and ALS patients. The CD4/CD8 ratio was normal in both groups and numbers of CD8^+^ cells, DN, DP, naïve and different types of memory T cells, as well as baseline HLA-DR^+^ cells, were comparable between both groups. Furthermore, we demonstrate that TDP-43 peptides induced a weak T-cell response, comparable to the control self-antigens actin and MOG. However, in contrast to PHA, actin and MOG, we found a statistically significant decrease in the activation of T cells from ALS patients by TDP-43. This suggests that although TDP-43 is a weak self-antigen, it still might be associated as such with ALS. One limitation of this study is the use of IL-2 for T-cell expansion, which has been shown to influence specifically regulatory T-cell function (34, 35). Thus, our data might be heavily influenced by the preferential expansion of regulatory T-cells in culture.

TDP-43 is a nucleo-cytoplasmic protein, which suggests the presentation of TDP-43 epitopes by the MHC-class I complexes. In addition, we have previously shown that TDP-43 oligomers can be released from cells into the extracellular space (27) and thus presentation by MHC-class II complexes should also be possible. In our experimental setup, cross-presentation of extracellular antigens by immature dendritic cells and possibly monocytes allows for the activation of both MHC-class I restricted and MHC-class II-restricted responses to the exogenously applied peptides. Indeed, we found both MHC-class I and MHC-class II-restricted weak antigen-specific responses, which implies that both cytotoxic and cytoprotective immune responses could be evoked by TDP-43. The presence of an MHC-class I-restricted T-cell response suggests cytotoxic effects on cells presenting TDP-43 antigens. Motor neurons express MHC-class I complexes in physiological as well as pathological conditions, like ALS (36). MHC-I also has a role in the stabilization of synapses and their recovery from injuries (37). The expression of MHC-class I is upregulated upon exposure to IFN-γ, also produced by activated cytotoxic T cells and activated microglia (38). In line with this, Coque et al. demonstrated recently that cytotoxic T cells can selectively induce the death of motoneurons in the SOD1^G93A^ mouse model (9). The presence of MHC-class II mediated responses, on the other hand, allows for cytoprotective immune responses. Interestingly, we found less activation of T cells by TDP-43 in ALS patients, suggesting a possible failure of cytoprotective T-cell mechanisms. Indeed, regulatory T cells are decreased in ALS and negatively correlate with disease progression (6). Thus, reduced T-cell responses to ALS-related antigens might be detrimental. In addition, the presence of antigen-reactive T cells is also an important requirement for the development of strong, successful humoral responses to antigens. Although T-cell-independent activation of antigen-specific B cells is possible, most antigens will require the simultaneous presence of an antigen-specific T-cell and B-cell for the development of strong humoral response. It is therefore conceivable that such antibody-mediated immune responses could halt the cell-to-cell transmission of TDP-43 pathology that we have previously demonstrated (27). In fact, a similar therapeutic strategy has already entered the clinical phase for alpha-synuclein in PD (39). Interestingly, we did not find a significant difference in the levels of TDP-43-specific auto-antibodies between ALS patients and controls. This is in disagreement with some previous studies, which found either elevation (40, 41) or a decrease (42) in naturally-occurring TDP-43 auto-antibodies in ALS. The discrepancy most likely stems from differences in methodology, as we employed a novel assay which allows the binding of the auto-antibodies to TDP-43 in liquid phase and includes monomeric, oligomeric and likely post-translationally modified TDP-43. Unfortunately, it was not possible in this study to correlate the levels of auto-antibodies to TDP-43 to the T-cell activation in the same patient, so this should be considered in future studies to elucidate better the relationship between T-cell activation by TDP-43 and the humoral response to it.

Importantly, the antigen-specific response to TDP-43 was very low across all conditions: the disease/control state, MHC-class I/II activation type, detection method, assayed cell types. Thus, our data does not directly provide evidence for the abovementioned processes in ALS. Further work is necessary to either identify other autoantigens in ALS or to demonstrate stronger TDP-43 responses in disease-relevant circumstances, like a strong activation of the innate immunity. The activation of innate immune cells is a prerequisite for a strong activation of the adaptive immune response. The TDP-43 peptides used in this study did not induce a significant activation of monocytes, supporting the specificity of the observed T-cell response (e.g. no contamination with endotoxin), but also likely reducing the magnitude of T-cell activation that would be seen if innate immune cells were also activated by TDP-43. Presentation by non-activated dendritic cells is an important mechanism for the induction of tolerance to an antigen, while presentation by activated dendritic cells will induce a strong T-cell response. Due to the ubiquity and importance of TDP-43 for the cell, tolerance to intracellular TDP-43 antigens is probably critical and reflected by the weak T-cell response and widespread reactivity we found also in healthy controls (~ 40% of donors), similarly to MOG and actin. Thus, we consider antigen presentation by PBMCs, as usual in the mixed-culture ELISPOT assay, sufficient for this model. However, future studies should consider the application of mature DCs as APCs to investigate the antigenic response to TDP-43 in full scale. Moreover, we speculate that the T-cell response to TDP-43 *in vivo* will be much stronger than what we observed in culture, if the source of TDP-43 antigens are *pathologic* forms of TDP-43 (oligomers, aggregates) that could also activate innate immune cells. Indeed, it is well-established that aggregated proteins may act as alarmins and induce a strong activation in APCs (43). In line with this, we have recently shown that not only aggregates, but also oligomers and mutant forms of alpha-synuclein can strongly enhance the activation of innate immune cells (44). The mounting of an adaptive immune response to abnormal TDP-43 in the CNS will require the presentation of TDP-43 antigens derived from CNS neurons by professional antigen-presenting cells (APC). Peripheral APCs will need to first collect the antigen from the CNS, a concept that was traditionally considered not possible unless there is some leakage from the blood-brain-barrier. However, recent studies have demonstrated that presentation of antigen from the CNS is possible in the glymphatic system and that peripheral lymphocytes may indeed circulate through the adult CNS. Alternatively, microglia and astrocytes may act as APCs in the CNS (45) and prime T cell for an immune response. In ALS, activated microglia are found throughout the affected CNS areas and express HLA-DR (2, 46, 47). Furthermore, studies have consistently reported infiltration of T cells into the CNS of ALS patients and an association with areas of degeneration (48, 49).

In summary, we show that human T cells bear the potential to recognize peptides derived from TDP-43. Taken together from our data and the current literature, the response is comparably weak, seen in a significant proportion of probands, may have role in tolerance, and could contribute to the neurodegenerative processes in ALS *in vivo.*

## Data availability statement

The raw data supporting the conclusions of this article will be made available by the authors, without undue reservation.

## Ethics statement

The studies involving human participants were reviewed and approved by Ethics Committee of Ulm University. The patients/participants provided their written informed consent to participate in this study.

## Author contributions

SR and BL performed the experiments, VG and MM analyzed the data, KK and SW collected samples, AL and JW provided intellectual input, VG, SR and KD wrote the manuscript. All authors reviewed and approved the manuscript.

## Supplementary material

Supplementary Figure 1 Circulating CD3+ T cells in ALS and healthy blood. **(A)** T cells were quantified based on the CD3 expression. CD3 subsets were quantified as % of their parent population, the total CD3^+^ cells. **(B)** Relative frequencies (as % of all CD3^+^ cells) of CD4^+^, CD8^+^, double-negative (DN) and double-positive (DP) T cells in CD3^+^ cells from healthy controls and ALS patients (n=25/24). Lines: median. *‘ns’*: not significant, Mann-Whitney U-test.

Supplementary Figure 2 Flow cytometry gating strategy for the quantification of T-cell activation by HLA-DR expression. **(A)** CD3^+^ T cells were selected from the total PBMCs and *HLA-DR* expression quantified as % of positive cells. CD4^+^ and CD8+ T cells were selected from the CD3^+^ cells and the *HLA-DR* expression quantified as % of positive cells. **(B)** Different memory subtypes were selected from the total CD3^+^ cells based on their expression of *CD45RA* and *CCR7* and *HLA-DR* expression quantified as % of positive cells in each subtype.

Supplementary Figure 3
*In-vitro* expansion of T-cell memory subsets by culture with IL-2 for 14 DIV. Relative proportions of the T-cell memory subsets in after 14 DIV with IL-2 treatment and no further stimulation. N = 9 (healthy controls). Lines: median. ***p<0.01*, ****p<0.001*, Mann-Whitney U-Test. DIV: Days *in vitro*. IL-2: 20 ng/mL.

Supplementary Figure 4 Monocyte activation by TDP-43 peptides. Peripheral blood monocytes were stimulated with 3 different concentrations of pooled TDP-43 peptides (1-10-100 µg/mL), LPS (1 ng/mL) or MOG (5 µg/mL) and IL-6 release quantified with ELISA. Lines: mean ± SEM, N = 3 healthy controls, 4 ALS patients.

Supplementary Figure 5 Antigen-restricted activation of T-cells by TDP-43 in responders. Activation of T cells was assessed with ELISPOT for IL-5 and IFN-γ after stimulation with TDP-43 peptides. Lines: median. *p<0.05, Mann-Whitney U-Test.

Supplementary Figure 6 T-cell memory to TDP-43 peptides. Activation of antigen-naïve and memory T cells by TDP-43 in all volunteers. N = 32 (healthy controls)/33 (ALS patients). Each data point is a single TDP-43 peptide in its mean T-cell response across all probands (healthy controls and ALS). Lines: median.

Supplementary Figure 7 Antibodies to α-synuclein in serum. Levels of antibodies to α-synuclein in serum from healthy controls and PD patients (n=22/22; 65.6 ± 11.4/68.8 ± 10.2 y; f/m: HC: 15/7, PD: 8/15) were measured with the same assay as auto-antibodies to TDP-43 in healthy control and ALS plasma. *‘ns’*: not significant (Mann-Whitney U-test). Serum of the one person with highest antibody levels was sampled on two different timepoints. Note the two segments of the Y-axis.
