# Supplementary material for: Low T-cell reactivity to TDP-43 peptides in ALS
Source: Front Immunol. 2023 Jul 21;14:1193507. doi: 10.3389/fimmu.2023.1193507 (PMC10401033; doi:10.3389/fimmu.2023.1193507)
Supplement: Supplementary Figure 1 — Circulating CD3+ T cells in ALS and healthy blood. (A) T cells were quantified based on the CD3 expression. CD3 subsets were quantified as % of their parent population, the total CD3+ cells. (B) Relative frequencies (as % of all CD3+ cells) of CD4+, CD8+, double-negative (DN) and double-positive (DP) T cells in CD3+ cells from healthy controls and ALS patients (n=25/24). Lines: median. ‘ns’: not significant, Mann-Whitney U-test. [file DataSheet_1.pdf]

Low T-cell reactivity to TDP-43 peptides in ALS

Swetha Ramachandran[1†], Veselin Grozdanov[1†], Bianca Leins[1], Katharina Kandler[1], Simon Witzel[1], Medhanie Mulaw[2], Albert C. Ludolph[1, 3], Jochen H. Weishaupt[4], Karin M. Danzer[1, 3*]

[1]Neurology, University Clinic, University of Ulm, Ulm, Germany
[2]Institute of Experimental Cancer Research, Medical Faculty, University of Ulm, Ulm, Germany
[3]German Center for Neurodegenerative Diseases (DZNE), Ulm
[4]Neurology, Medical Faculty Mannheim, Heidelberg University, Heidelberg, Germany

* Correspondence:

Prof. Dr. Karin M. Danzer

E-Mail: karin.danzer@dzne.de

Supplementary Tables

	Control	ALS
Number	25	24
Age	61.67 ± 11.83	60.37 ± 11.77
Gender (f/m)	12/13	5/19
Disease duration from onset (months)	NA	26.87 ± 22.56
Disease duration from diagnosis (months)	NA	18.27 ± 18.96
ALSFRS-R	NA	33.26 ± 9.54
sALS/ fALS	NA	20/4
Riluzole (y/n)	NA	20/4

Suppl. Table 1. Cohort characteristics (related to Fig. 1). Age, disease duration and ALSFRS-R: mean ± standard deviation. ALSFRS-R: Revised Amyotrophic Lateral Sclerosis Functional Rating Scale. sALS/fALS: Sporadic ALS/familial ALS.

	Control	ALS
**Number**	9	9
**Age**	55.56 ± 11.52	57.97 ± 9.93
**Gender (f/m)**	3/6	1/8
**Disease duration from onset (months)**	NA	21.11 ± 20.29
**Disease duration from diagnosis (months)**	NA	15.33 ± 19.59
**ALSFRS-R**	NA	31.22 ± 11.96
**sALS/ fALS**	NA	9/0
**Riluzole (y/n)**	NA	9/0

Suppl. Table 2. Cohort characteristics (related to Fig. 2). Age, disease duration and ALSFRS-R: mean ± standard deviation. ALSFRS-R: Revised Amyotrophic Lateral Sclerosis Functional Rating Scale. sALS/fALS: Sporadic ALS/familial ALS.

**Sequence**	**Peptide Length**	**MHC typeStarting position**	**Starting position**	**Ending position**
**AQFPGACGLRY**	11	I	33	43
**GWGNLVYVVNYPKDN**	15	II	67	89
**ASSAVKVKR**	9	I	90	98
**TSDLIVLGLPWKTTE**	15	II	103	117
**STFGEVLMV**	9	I	125	133
**KVMSQRHMI**	9	I	160	168
**ELREFFSQY**	9	I	206	214
**VTFADDQIAQSLCGE**	15	II	232	246
**GMNFGAFSINPAMMA**	15	II	310	324
**NGGFGSSMDSKSSGW**	15	II	398	412

Suppl. Table 3. TDP-43 peptides for the stimulation of T cells.

	Control	ALS
**Number**	32	33
**Age**	63.78 ± 11.82	62.12 ± 9.83
**Gender (f/m)**	13/19	8/25
**Disease duration from onset (months)**	NA	48.96 ± 37.5
**Disease duration from diagnosis (months)**	NA	31.6 ± 30.4
**ALSFRS-R**	NA	32.25 ± 10.9
**sALS/fALS**	NA	32/1
**Riluzole (y/n)**	NA	29/4

Suppl. Table 4. Cohort characteristics (related to Fig. 3). Age, disease duration and ALSFRS-R: mean ± standard deviation. ALSFRS-R: Revised Amyotrophic Lateral Sclerosis Functional Rating Scale. sALS/fALS: Sporadic ALS/familial ALS.

		**HC, Mean±SD**	**ALS, Mean±SD**	**HC, Median(IQR)**	**ALS, Median(IQR)**	**p-value**	**signif.**
**IL-5**	**PHA**	290±213	266±257	266(112-420)	128(71-472)	0.45	ns
	**Actin**	19±30	18±50	4(1-20)	3(1-11)	0.32	ns
	**MOG**	12±25	26/65	1(0-8)	3(0-10)	0.79	ns
	**TDP-43**	13±20	6±10	3(1-20)	2(0-7)	0.03	*
**IFN-γ**	**PHA**	223±235	153±226	168(13-355)	61(18-159)	0.21	ns
	**Actin**	15±44	4±10	1(0-4)	1(0-4)	0.87	ns
	**MOG**	5±11	9±35	0(0-2)	0(0-3)	0.86	ns
	**TDP-43**	9±24	3±6	1(0-5)	1(0-3)	0.38	ns

Suppl. Table 5. Summary statistics of ELISPOT results. SD: standards deviation (of the sample); IQR: interquartile range; *'ns'*: not significant. All differences tested with Mann-Whitney U-test.

**Peptide**	**HC, Mean±SD**	**ALS, Mean±SD**	**HC, Median (IQR)**	**ALS, Median (IQR)**	**p-value**	**Sign.**

**33-43**	18±30	6±9	4(1-14)	1(0-8)	0.35	ns
**67-89**	9±17	2±4	2(1-13)	1(0-3)	>0.99	ns
**90-98**	17±28	6±14	3(1-16)	1(0-6)	0.81	ns
**103-117**	6±10	3±5	1(0-5)	1(0-5)	>0.99	ns
**125-133**	4±8	3±6	1(0-5)	1(0-4)	>0.99	ns
**160-168**	17±31	6±16	5(0-18)	1(0-5)	0.78	ns
**206-214**	8±21	3±6	1(0-7)	1(0-3)	>0.99	ns
**232-246**	21±55	9±19	2(0-15)	1(0-9)	0.41	ns
**310-324**	24±58	8±14	2(0-16)	2(0-7)	0.06	ns
**398-412**	8±12	11±16	2(0-9)	2(1-13)	>0.99	ns

Suppl. Table 6. Summary statistics of ELISPOT IL-5 results for stimulation with single TDP-43 peptides. SD: standards deviation (of the sample); IQR: interquartile range; *'ns'*: not significant. All differences tested with one-way ANOVA with multiple comparisons with Bonferroni's correction.

**IFNg**						
**Peptide**	**HC, Mean±SD**	**ALS, Mean±SD**	**HC, Median (IQR)**	**ALS, Median (IQR)**	**p-value**	**Sign.**
**33-43**	9±24	3±8	1(0-4)	1(0-2)	>0.99	ns
**67-89**	4±9	1±4	1(0-3)	0(0-1)	>0.99	ns
**90-98**	10±29	3±6	1(0-3)	1(0-2)	>0.99	ns
**103-117**	2±5	2±3	0(0-1)	0(0-2)	>0.99	ns
**125-133**	1±2	1±2	0(0-2)	0(0-1)	>0.99	ns
**160-168**	9±34	2±4	1(0-2)	0(0-2)	>0.99	ns

**206-214**	7±21	7±29	0(0-4)	1(0-3)	>0.99	ns
**232-246**	22±69	5±11	1(0-7)	1(0-4)	0.08	ns
**310-324**	24±64	5±10	1(0-9)	1(0-4)	0.03	*
**398-412**	6±21	3±5	1(0-3)	1(0-3)	>0.99	ns

Suppl. Table 7. Summary statistics of ELISPOT IFN-γ results for stimulation with single TDP-43 peptides. SD: standards deviation (of the sample); IQR: interquartile range; *'ns'*: not significant, **p<0.05*. All differences tested with one-way ANOVA with multiple comparisons with Bonferroni's correction.
