# Supplementary figures and images for: Low T-cell reactivity to TDP-43 peptides in ALS
Source: Front Immunol. 2023 Jul 21;14:1193507. doi: 10.3389/fimmu.2023.1193507 (PMC10401033; doi:10.3389/fimmu.2023.1193507)

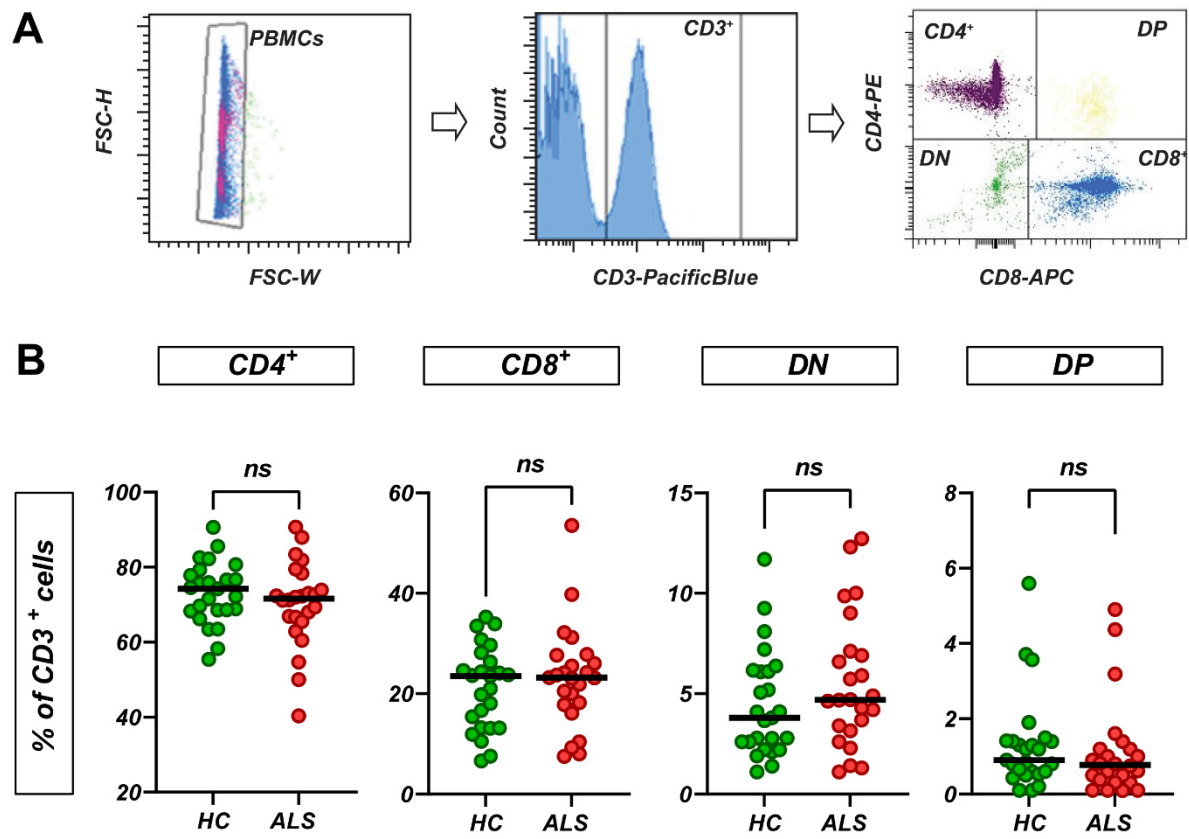

Suppl. Fig. 1

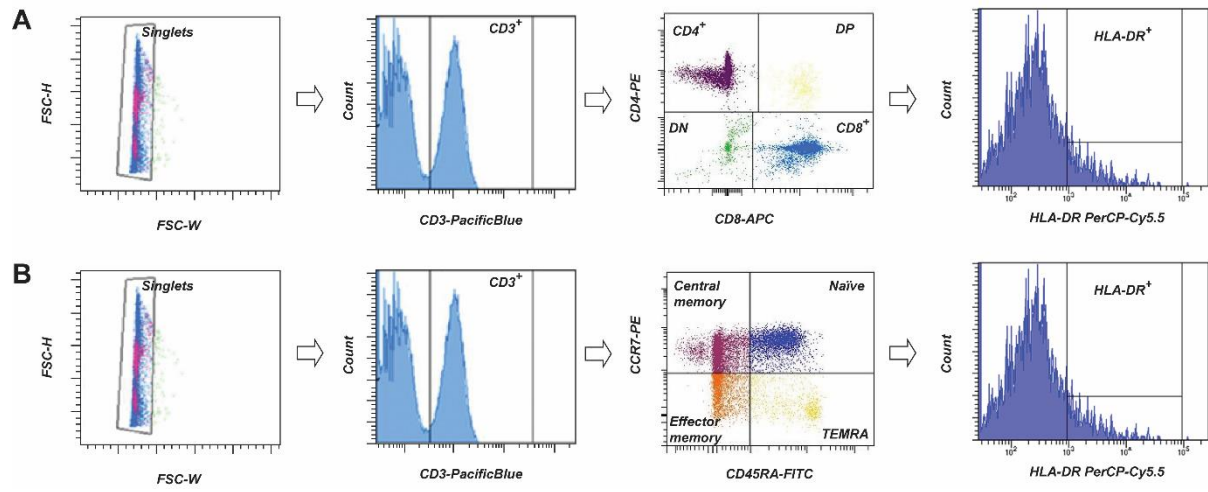

Suppl. Fig .2

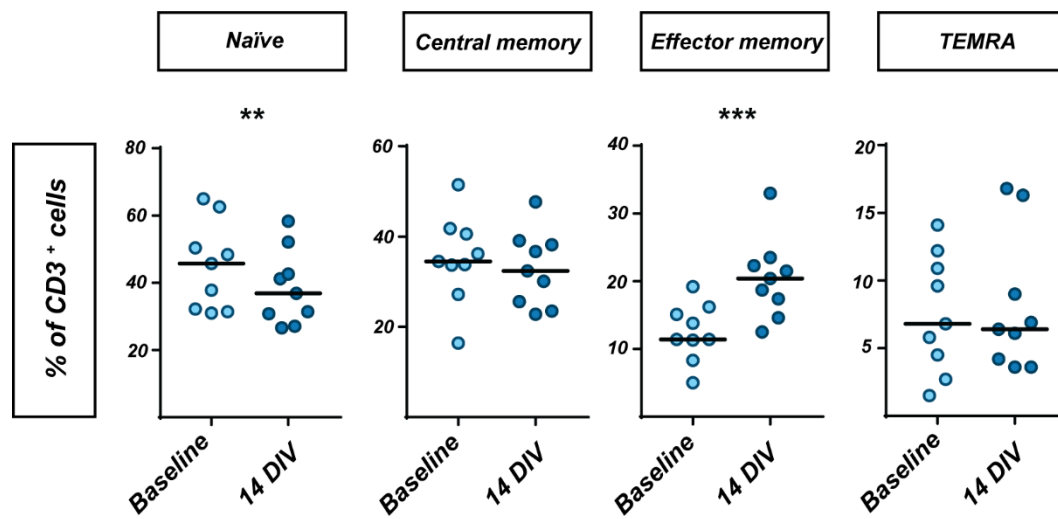

Suppl. Fig. 3

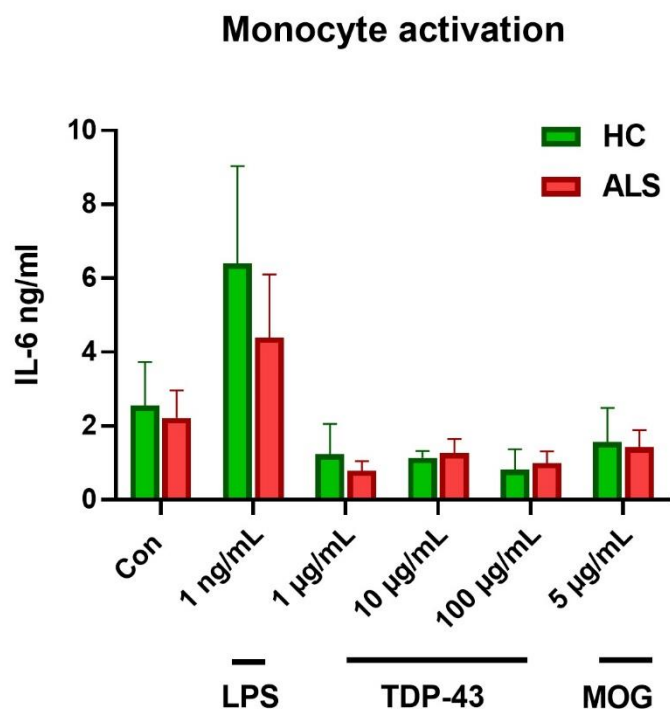

Suppl. Fig. 4

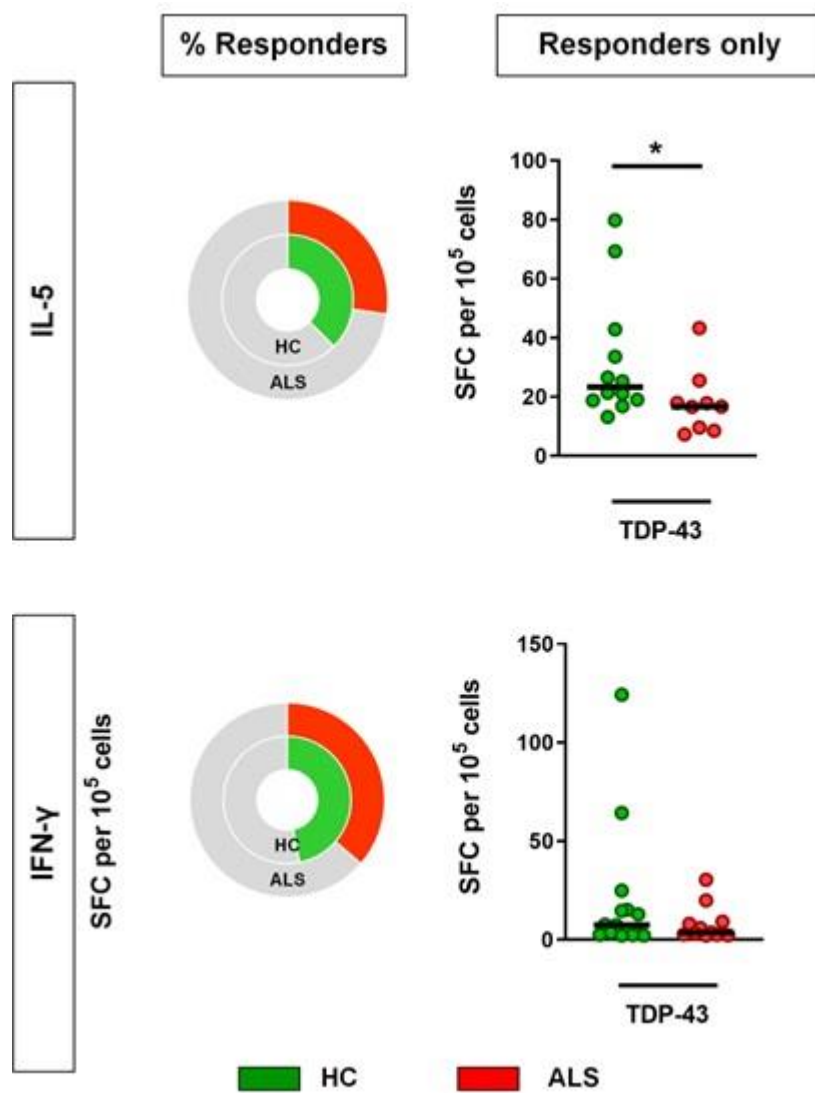

Suppl. Fig. 5

IL-5

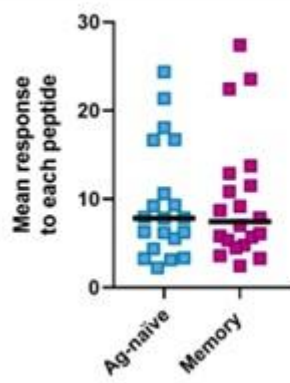

IFN- $\gamma$

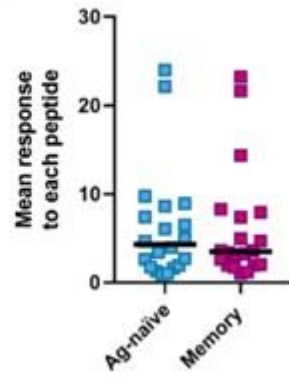

Suppl. Fig. 6

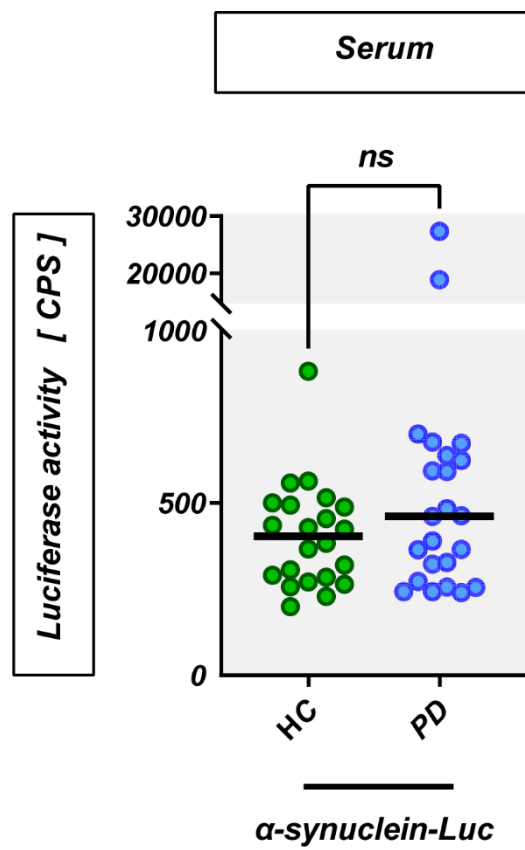

Suppl. Fig. 7

Supplement: Supplementary file 2 [file DataSheet_2.pdf]
